# Steps To Prevent Mortality in a Patient with Coinciding Severe Sepsis and Cardiogenic Shock Post-Non-ST-Elevation Myocardial Infarction (NSTEMI): A Case Report

**DOI:** 10.7759/cureus.32086

**Published:** 2022-11-30

**Authors:** Julius M Nagaratnam, Rubab Farooq, Mirna El Dirani, Shaun Mathew, Celestine I Ogwu, Samer Kholoki

**Affiliations:** 1 Internal Medicine, Avalon University School of Medicine, Chicago, USA; 2 Internal Medicine, Saint James School of Medicine, Chicago, USA; 3 Internal Medicine, Washington University of Health and Science, Chicago, USA; 4 Internal Medicine, Richmond Gabriel University, Chicago, USA; 5 Internal Medicine, La Grange Memorial Hospital, Chicago, USA

**Keywords:** acute kidney injury, cardiogenic shock, severe sepsis, nephrolithiasis, non-st-elevation myocardial infarction (nstemi)

## Abstract

Severe sepsis is characterized by acute organ dysfunction secondary to an infective source, often requiring emergent medical intervention. The severity of sepsis is determined by a criterion that focuses on the presence of fever, tachycardia, tachypnea, leukocytosis, lactic acidosis, hypotension, evidence of organ failure, and the presence of an infective source. Management of sepsis in patients with a coinciding ischemic event such as a myocardial infarction (MI), is difficult, given the prognosis is poor and there is a high risk for mortality. This case report explores methodical medical measures taken to prevent mortality in an 81-year-old Hispanic male that developed severe sepsis in conjunction with a complicated presentation of a non-ST-elevation myocardial infarction (NSTEMI).

## Introduction

Sepsis is routinely grouped and managed based on the causal pathogen, whether that may be bacterial, viral, fungal, or parasitic [1]. Bacteria are the most common causes of infection with 62.2% being attributed to gram-negative bacteria, and 46.8% being attributed to gram-positive bacteria [1]. The source of sepsis is most commonly due to infection of the respiratory system, such as pneumonia, followed by infection of the genitourinary system and other abdominal sources [2]. Failure to manage sepsis with early intervention via the use of antimicrobials may often lead to the progression of complications such as severe sepsis, septic shock, and toxic encephalopathy [2,3]. Early intervention of sepsis is particularly important to prevent mortality in cases with additional emergent morbidities such as the presence of coinciding cardiac ischemia.

A myocardial infarction (MI) results from a sudden interruption in blood flow in one or more coronary arteries, decreasing oxygen supply to a part of the heart muscle, and leading to necrosis and release of cardiac biomarkers (e.g., troponins) [4]. The types of MI are further categorized into an ST-elevation myocardial infarction (STEMI), and an NSTEMI, which are dependent on the findings of an electrocardiogram (ECG) [5]. Decreased heart function secondary to cardiac muscle death results in decreased blood flow and oxygen delivery to other organ systems, leading to complications such as acute kidney injury (AKI), a risk factor for inpatient mortality [6]. In addition, a fatal complication that follows 5% to 10% of MIs is the occurrence of cardiogenic shock, which is the most common cause of death post-MI [7].

Cardiogenic shock is often characterized by the criteria used in the (SH)ould we emergently revascularize (O)ccluded (C)oronaries for cardiogenic shoc(K) (SHOCK) trial. The criteria include a systolic blood pressure <90mmHg or the requirement of vasopressors to maintain systole >90mmHg; evidence of end-organ damage like urine output <30ml/h or the presence of cool extremities; and a cardiac index (CI) <2.2L/min/m2 or a pulmonary capillary wedge pressure >15mmHg [8]. A trial conducted between 2004 and 2006 analyzed the risk of mortality in patients undergoing percutaneous coronary intervention (PCI) post-MI after receiving pexelizumab versus placebo and demonstrated similar rates of death, shock, and heart failure between both groups [9]. Another study conducted in 2012, using the same database of patients, demonstrated that patients with severe infection post-MI were associated with a significantly higher rate of death (hazard ratio of 5.6, 95% confidence interval 3.8-8.4) [10]. The poor prognosis outlined by these studies warrants the need for methodical and immediate medical decision-making when managing such complicated cases.

## Case presentation

On the 24th of June 2021 (day one), an 81-year-old Hispanic male presented to the emergency department with a one-week history of dyspnea, weakness, and lethargy. Four days prior to his admission, the patient sustained a fall at his home and was placed back in his bed where he remained immobile. During this period, he had reduced oral intake of food and liquids, subsequently reduced urine output and bowel movements, and the patient became weaker, resulting in his admission. The patient has a past medical history significant for type II diabetes mellitus, hypertension, hyperlipidemia, benign prostatic hyperplasia (BPH), stage IV chronic kidney disease (CKD) (glomerular filtration rate = 26.8 ml/min/1.73m2), recurrent urinary tract infections (UTIs), bilateral knee osteoarthritis and lumbago with sciatica, and class I obesity (body mass index = 33.6 kg/m2). He denied having a history of tobacco, alcohol, or other forms of substance abuse, and having a prior MI or stroke. His home medication list consisted of Tylenol 325mg as needed (PRN), Norco 10 mg/325 mg PRN, alfuzosin 10 mg daily (QD), allopurinol 100 mg QD, aspirin 81 mg QD, celecoxib 200 mg QD, diltiazem 240 mg QD, gemfibrozil 600 mg twice daily (BID), metformin 1000 mg QD, prednisone 10 mg QD, simvastatin 40 mg QD, and tamsulosin 0.4 mg QD.

In addition, the patient reported having body aches, intermittent fever, and chills since sustaining the fall, as well as dysuria. The patient denied having a cough, urinary frequency, urgency, chest pain, or other associated symptoms. Physical examination findings were significant for a fatigued and pale male that was alert and oriented to person, place, and time, with weak bilateral dorsalis pedis and posterior tibial pulses, and the presence of a Dilaudid pump positioned on his lower back for management of his osteoarthritis. There were no other abnormal physical findings. Vitals demonstrated that the patient was hypotensive (82/50 mmHg) and febrile (100°F), however, his remaining vitals were within normal limits. Laboratory findings on admission demonstrated leukocytosis with a white blood cell (WBC) count of 15,400 cells/mm3, troponin of 4544 ng/L which increased to 7358 ng/L six days after whilst admitted, brain natriuretic peptide of 514 pg/mL, creatinine of 2.64 mg/dL, lactate of 2.8 mmol/L, and a creatine phosphokinase (CPK) of 272 units/L.

Urine analysis done on arrival demonstrated pyuria and hematuria, and a urine and blood culture obtained positive readings for gram-negative rods and *Proteus mirabilis* 48 hours after arrival. Chest X-ray demonstrated interstitial markings suggestive of moderate edema or congestion, and diffuse opacities in the right and left hemithorax. The ECG on admission demonstrated normal sinus rhythm with premature atrial complexes, non-specific ST changes, and a left-axis deviation. The patient’s global registry of acute coronary events (GRACE) score was 204 points, and the thrombolysis in myocardial infarction (TIMI) score was 7 points. The patient met four of the criteria for severe sepsis [7], requiring transfer to the intensive care unit (ICU), placement on intravenous (IV) fluids, and cefepime. The infectious disease team was consulted, and plans for a peripherally inserted central catheter (PICC) were made for high-volume antibiotic administration once WBC levels decreased. The patient’s findings were also consistent with the findings of an NSTEMI and he was scheduled for a transthoracic echocardiogram (TTE) and cardiac catheterization when hemodynamically stable. The patient was also placed on a medication regimen of IV norepinephrine, famotidine 20 mg, hydrocortisone 50 mg, oral atorvastatin 40 mg QD, subcutaneous insulin aspart, and heparin 5000 units every eight hours. From his home medication list, only allopurinol, celecoxib, gemfibrozil, and tamsulosin were initially continued.

The TTE performed on day 2 of admission demonstrated an ejection fraction of 35% to 40%, mildly enlarged left ventricle (6.1 cm), mildly dilated left and right atrium, moderate anterior wall hypokinesis and aortic regurgitation, pulmonary artery pressure of 45 mmHg, and a right atrial pressure of 20 mmHg. The confidence interval (CI) was found to be 1.97 L/min/m2, which confirmed that the patient met all three parts of the criteria for cardiogenic shock.

On day three the patient became hemodynamically stable, was taken off IV norepinephrine and transferred to the progressive care unit, and was switched from IV cefepime to cefazolin. However, he became increasingly confused, being only oriented to person and place, and was unable to recall past events, which required a head computerized tomography (CT) without contrast. Findings showed no focal neurologic deficits, ruling the confusion due to toxic-metabolic encephalopathy.

The patient denied being able to void adequate urine, and hence on day four a bladder scan was performed obtaining a post-void residual volume of 900 ml. A renal ultrasound was also performed, which demonstrated mild right-sided hydronephrosis, and a foley catheter was inserted to improve urine flow. On day five, the patient underwent a coronary angiogram which demonstrated total occlusion of the left anterior descending artery at the middle segment with faint anterograde filling distally. The distal filling was explained by the presence of left-to-left and right-to-left collateral arteries. There was also a moderate disease of the obtuse marginal artery 1 branching of the left circumflex artery. Post-catheterization interventions like PCI and balloon angioplasty were avoided, and recommendations were to manage the patient with a regimen of clopidogrel 75 mg QD, aspirin, and metoprolol 25 mg QD. 

**Figure 1:**
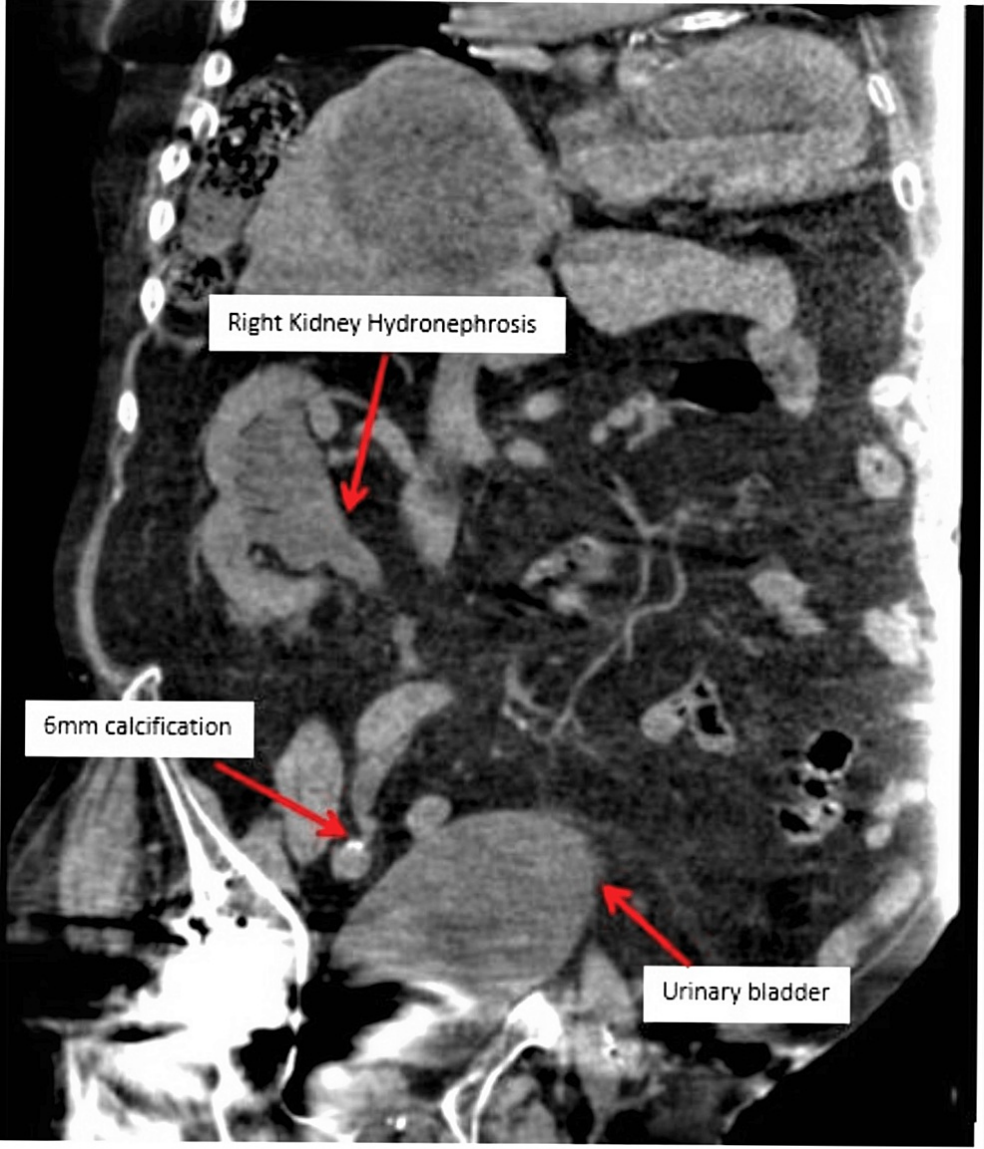
Abdominal CT demonstrating right kidney hydronephrosis and distal 6 mm calcification

**Figure 2:**
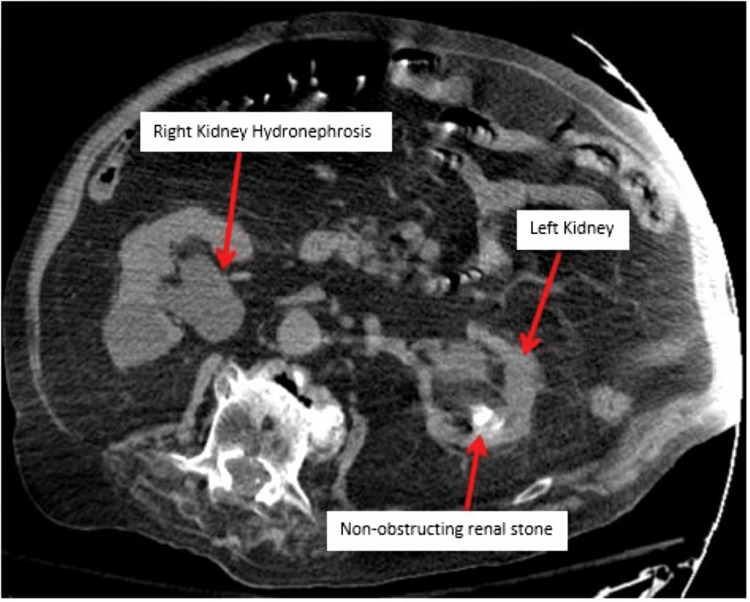
Abdominal CT demonstrating right kidney hydronephrosis, and non-obstructive renal stone in left kidney

On day seven, a PICC was placed. An abdominal CT performed on day eight demonstrated right-sided hydronephrosis with a 6 mm opacity at the distal aspect of the ureter (Figure 1), as well as the presence of non-obstructing renal calculus in the left calyx (Figure 2). The patient underwent a cystoscopy and right ureteral stent placement on day 14, which improved the patient’s signs and symptoms. Eventually, the patient’s PICC and foley catheter were removed and he was discharged to a skilled nursing facility on day 16 with his original medication list plus torsemide 10 mg QD, finasteride 5 mg QD, carvedilol 6.25 mg QD for improvement of systolic left ventricular dysfunction, and cephalexin 500 mg four times a day to BID taper.

## Discussion

In this case, we observed multiple acute comorbidities in the form of severe sepsis secondary to a UTI and nephrolithiasis, AKI secondary to an NSTEMI, and cardiogenic shock also secondary to an NSTEMI, which warranted a poor prognosis in this patient. Also factoring in chronic comorbidities such as class I obesity, hypertension, hyperlipidemia, type II diabetes, pre-existing BPH, and stage IV CKD, as well as being of advanced age, suggests a low probability of survival [11]. However, despite the patient’s complicated medical history, we can attribute his survival to correct methodical medical management.

NSTEMI and cardiogenic shock management

Initial management in acute coronary syndromes (ACS) such as an NSTEMI involves assessing the severity of a patient’s presentation using a Global Registry of Acute Coronary Events (GRACE) score [12]. The GRACE score utilizes the patient's systolic blood pressure, age, heart rate, cardiac enzymes, creatinine levels, ECG findings, any events of cardiac arrest on admission, and Killip classification, to determine the risk of mortality within six months after admission [12]. Patients with a low-risk GRACE score (<109 points) are usually treated via pharmacotherapy such as aspirin, anticoagulants, beta-blockers, nitroglycerin, and statins [13,14]. Patients with high-risk GRACE scores (>140 points) are treated with a PCI or a coronary artery bypass graft (CABG) formation within 24 hours after admission as well as pharmacotherapy [13,14]. Patients with an intermediate-risk GRACE score (109-140 points) are candidates for PCI placement or CABG formation within 25 to 72 hours after admission [13,14].

The TIMI score is another mode of measuring the risk of mortality in patients with an NSTEMI or unstable angina [12,13]. The TIMI score utilizes three risk factors for ACS as well as the use of aspirin within seven days to calculate a patient’s risk of mortality [15]. Management is relative to the value of the TIMI score, with scores of 1 to 2 points being characterized as being low risk, scores of 3 to 5 points being characterized as intermediate risk, and scores of 6 to 7 points being characterized as high risk [15]. Immediate utilization of both scoring systems is imperative for early intervention, and to prevent progression to poorly prognostic conditions such as cardiogenic shock and eventual death.

However, in circumstances where the patient progresses to cardiogenic shock, immediate use of the SHOCK criteria is fundamental to prevent mortality. The use of vasopressors, IV fluids, and pharmacotherapy with positive inotropic activity is the first line of management for cardiogenic shock [16], and then further minimally invasive procedures can be undertaken once the patient is hemodynamically stable.

AKI, nephrolithiasis, and severe sepsis management

Cardiac ischemia leading to prerenal AKI is an inevitable complication. Common laboratory findings in patients with AKI include elevated blood urea nitrogen (BUN) and creatinine levels, which are indicative of impaired renal absorption and secretion [17]. Acute kidney injury is traditionally conservatively managed through the replenishment of IV fluids and the avoidance of nephrotoxic medication such as non-steroidal anti-inflammatory drugs (NSAIDs), and the diabetic medication, metformin [18]. However, unlike most other cases, this patient’s AKI was further complicated with nephrolithiasis associated with a UTI.

The patient’s AKI dramatically impacts renal filtration [6] and increases stone precipitation. In addition, this patient’s UTI was secondary to *Proteus* infection which is associated with the formation of large renal stones referred to as staghorn calculi. The formation of renal stones often causes ureteral obstruction which is associated with hydronephrosis, a form of post-renal injury that exacerbates the patient’s AKI. Fortunately, this patient did not have staghorn calculi present on imaging, however, he did have multiple smaller stones, in particular, a right-sided obstructing stone. Smaller stones can be managed through techniques such as shock wave lithotripsy, and ureteroscopy [19]. The use of appropriate imaging such as renal ultrasounds and abdominal CTs is fundamental for determining the appropriate form of management and for preventing the progression to sepsis, which is further associated with complications such as septic shock, and toxic encephalopathy [3].

In this case, however, the patient progressed to severe sepsis due to delayed management of his UTI and nephrolithiasis. Prognostically, the patient had a poor chance of survival given his recent MI and cardiogenic shock. However, via the use of a PICC line for the administration of high-volume antibiotics, the patient’s leukocytosis and infection were controlled over a short period. In addition, immediate localization of the infectious source (i.e., the presence of UTI and nephrolithiasis), was fundamental for rapid control of his infectious state, and prevention of progression into septic shock.

## Conclusions

Scoring systems such as the GRACE and TIMI scores, as well as appropriate imaging, are essential for determining the next steps in medical management in NSTEMI patients with complicated presentations. Downstream complications such as AKI are managed conservatively and urgently via IV fluids and avoidance of nephrotoxic medication. Further localization of the infectious source is fundamental in controlling the patient’s sepsis and prevention of progression. Obtaining hemodynamic stability, and then immediately localizing the source of infection was the main reason for preventing mortality in our patient. This decision-making creates great emphasis on the importance of methodical medical management.
